# In Vitro Antibacterial Activity of Green Synthesized Silver Nanoparticles Using *Mangifera indica* Aqueous Leaf Extract against Multidrug-Resistant Pathogens

**DOI:** 10.3390/antibiotics11111503

**Published:** 2022-10-28

**Authors:** Yahya S. Alqahtani, Amal Bahafi, Kiran K. Mirajkar, Rakshith Rudrapura Basavaraju, Susweta Mitra, Shailaja S, Sunil S. More, Uday M. Muddapur, Aejaz Abdullatif Khan, P. Renuka Sudarshan, Ibrahim Ahmed Shaikh

**Affiliations:** 1 Department of Pharmaceutical Chemistry, College of Pharmacy, Najran University, Najran 66462, Saudi Arabia; 2 Department of Pharmaceutical Chemistry, Ibn Sina National College for Medical Studies, Jeddah 21418, Saudi Arabia; 3 Department of Biochemistry, University of Agricultural Sciences, Dharwad 580005, India; 4 School of Basic and Applied Sciences, Dayananda Sagar University, Bangalore 560111, India; 5 Department of Biotechnology, KLE Technological University, BVB Campus, Hubli 580031, India; 6 Department of General Science, Ibn Sina National College for Medical Studies, Jeddah 21418, Saudi Arabia; 7 Department of Pharmacology, College of Pharmacy, Najran University, Najran 66462, Saudi Arabia

**Keywords:** MDR pathogens, silver nanoparticles, antibacterial activity, *Mangifera indica* leaf extract, *Triticum* spp.

## Abstract

An estimated 35% of the world’s population depends on wheat as their primary crop. One fifth of the world’s wheat is utilized as animal feed, while more than two thirds are used for human consumption. Each year, 17–18% of the world’s wheat is consumed by China and India. In wheat, spot blotch caused by *Bipolaris sorokiniana* is one of the major diseases which affects the wheat crop growth and yield in warmer and humid regions of the world. The present work was conducted to evaluate the effect of green synthesized silver nanoparticles on the biochemical constituents of wheat crops infected with spot blotch disease. Silver nanoparticles (AgNPs) were synthesized using *Mangifera indica* leaf extract and their characterization was performed using UV-visible spectroscopy, SEM, XRD, and PSA. Characterization techniques confirm the presence of crystalline, spherical silver nanoparticles with an average size of 52 nm. The effect of green synthesized nanoparticles on antioxidative enzymes, e.g., Superoxide dismutase (SOD), Catalase (CAT), Glutathione Reductase (GR), Peroxidase (POX), and phytochemical precursor enzyme Phenylalanine Ammonia-Lyase (PAL), and on primary and secondary metabolites, e.g., reducing sugar and total phenol, in *Bipolaris sorokiniana* infected wheat crop were studied. Inoculation of fungal spores was conducted after 40 days of sowing. Subsequently, diseased plants were treated with silver nanoparticles at different concentrations. Elevation in all biochemical constituents was recorded under silver nanoparticle application. The treatment with a concentration of nanoparticles at 50 pp min diseased plants showed the highest resistance towards the pathogen. The efficacy of the green synthesized AgNPs as antibacterial agents was evaluated against multi drug resistant (MDR) bacteria comprising Gram-negative bacteria *Escherichia coli* (*n* = 6) and *Klebsiella pneumoniae* (*n* = 7) and Gram-positive bacteria Methicillin resistant *Staphylococcus aureus* (*n* = 2). The results show promising antibacterial activity with significant inhibition zones observed with the disc diffusion method, thus indicating green synthesized *M. indica* AgNPs as an active antibacterial agent against MDR pathogens.

## 1. Introduction

Wheat (*Triticum* spp.), the so-called “King of cereals*”* is considered one of the oldest staple food crops grown worldwide, with an annual production of 734 million tonnes [1]. Cultivation of wheat started as a part of the ‘Neolithic Revolution’ 10,000 years ago. The wheat kernel contains an average of 12% moisture, 70% carbohydrates, 12% proteins, 2% fat, 2.2% crude fibre, and 1.8% minerals. Niacin, riboflavin, and thiamine are the major vitamins present in wheat [2]. *Triticum aestivum, T. durum, T. dicoccum,* and *T. sphaerococcum* are the four important wheat species grown globally. Agriculture production is greatly affected by abiotic and biotic stresses, leading to huge economic losses. Spot blotch disease of wheat caused by *Bipolaris sorokiniana* is one of the major diseases which causes severe yield loss in wheat crop grown in the warm and humid regions of the world [3]. Although this disease was reported in the early 1990s, its importance was recognized only after the green revolution, when more numbers of semi-dwarf wheat cultivars became susceptible to spot blotch. When a crop reaches the late post-anthesis stage, which coincides with warm and high humid conditions, disease severity is higher.

Nanomaterials are used efficiently for the safe administration of pesticides, herbicides, and fertilizers at low concentrations. Pesticides affect human health and pollinating insects. To reduce the harmful effects of these chemicals, the use of green nanoparticles can be beneficial. Green nanoparticles play an important role in decreasing toxicity and, in turn, increase efficiency [4,5].

Consequently, new techniques are implemented by the modern agriculture system to minimize yield losses by several crop diseases. Among these technologies, nanotechnology has assumed a prominent place by virtue of its vast range of applications in various fields, e.g., agriculture, pharmaceuticals, electronics, etc. The development and application of biosynthesized nanotechnology in agricultural research initiated the development of an eco-friendly and effective biosynthesized nanoparticles to control diseases. The biological manufacture of silver nanoparticles (AgNPs) has been demonstrated as a reliable, nontoxic, and environmentally acceptable strategy for plant disease treatment [6]. Previous research has demonstrated the toxicity of silver nanoparticles on the growth of fungal hyphae and conidia, while AgNPs made from cow milk have also shown potent antifungal action against a variety of phytopathogens.

Plants contain a multitude of structurally divergent phytochemicals which are together termed as secondary metabolites. These metabolites act as natural pesticides, antibiotics and protective agents which are normally toxic to microbes. Plants can activate different metabolic pathways to control pathogen attacks. Phytoalexins, lignin, and phytoanticipins are the different compounds produced by the plant to overcome pathogen attacks. Biochemical resistance against diseases depends on pre-existing and induced substances present in the plant. Elicitors are the prime molecules capable to induce defense responses. Both exogenous (pathogen origin) and endogenous (plant origin) elicitors act as defense inducers [7]. The programmed cell death mechanism is a complex defense system shown by the plant to overcome the pathogen attack. Superoxide anion (O_2_^−^), hydroxyl radical (OH•) and Hydrogen peroxide (H_2_O_2_) are the major reactive oxygen species (ROS) produced by the plant under these conditions and are strong oxidizing agents which attack all the biomolecules causing cellular damage. Plant cells contain oxygen radical detoxifying enzymes, e.g., catalase, peroxidase, superoxide dismutase, ascorbate peroxidase, and glutathione reductase, and non-enzymatic antioxidants, such as ascorbic acid and phenolic derivatives, to overcome the ROS.

The SOD, which catalyzes the dismutation of superoxide anion to hydrogen peroxide (H_2_O_2_), appears to play an important role in the emergence and progression of the necrotic reaction. The induction of SOD activity in plants has been widely observed in response to pathogen invasion, and it is a reaction that is frequently related with plant resistance. Catalase is the most important enzyme in the elimination of hydrogen peroxide. In addition to serving as a substrate for the POD and CAT, the hydrogen peroxide produced by the SOD plays an important function in the synthesis of lignin [8]. Furthermore, due to its diffusible nature, hydrogen peroxide increases the expression of defense genes, such as phenylalanine ammonia-lyase, chalcone synthase, endochitinases, and phytoalexin biosynthetic enzymes, and is a major indicator of the related necrotic reaction. The hydrogen peroxide also induces the expression of genes that code for antioxidant proteins, such as glutathione-S-transferase, glutathione peroxidase, and polyubiquitins, which prevent the death of healthy cells with hypersensitivity [9,10]. The phenylpropanoid pathway leads to the biosynthesis of a wide range of phenolics. In many circumstances, the activation of the phenylpropanoid metabolism, with the main enzyme phenylalanine ammonia-lyase (PAL), is involved in the initial disease resistance reactions of plants, leading to the creation of several defense-related chemicals such as antimicrobial phytoalexins and lignin [11]. PAL has been linked to barley and wheat defense responses to the necrotrophic fungal disease caused by *Bipolaris sorokiniana* (Sacc.) [12]. After inoculating barley and wheat leaves with highly or weakly aggressive isolates of *Bipolaris sorokiniana*, PAL activity was strongly induced [13].

The main objective of this study was to evaluate the effect of green synthesized *M. indica* AgNPs on antioxidative enzymes and secondary metabolites, such as reducing sugar and total phenol, in *Bipolaris sorokiniana* infected wheat crop.

## 2. Materials and Methods

### 2.1. Source of Plant Material (Mangifera indica) and Chemicals

*M. indica* fresh leaves were collected, thoroughly washed several times with tap water followed by distilled water to remove the dust particles, and air-dried at room temperature for six to seven days. Dried leaves powder was prepared and stored at room temperature for further use. In this study, standard biochemical reagents and chemicals, including potassium phosphate, H_2_O_2_, AgNO_3_, etc., were of analytical grade, procured from Sigma Aldrich, Himedia, and Sisco Research Laboratories Pvt. Ltd.

### 2.2. Preparation of the M. indica Aqueous Leaf Extract

The aqueous *M. indica* leaf extract was prepared following the protocol described by Narayana et al. with minor modifications. Thus, 10 g of *M. indica* leaves powder was boiled in 100 mL of distilled water for 30 min (10% aqueous plant extract) and filtered through Whatman No. 1 filter paper. The filtrate was collected and stored in the refrigerator at 4 °C until further use.

### 2.3. Green Synthesis of Silver Nanoparticles (AgNPs) Using M. indica Aqueous Leaf Extract

The AgNO_3_ solution was used as a precursor for the synthesis of silver nanoparticles (AgNPs). Hence, 0.02 M solutions were prepared by the addition of 1.7 g of AgNO_3_ to 500 mL of distilled water. For the green synthesis of AgNPs we used 0.02 M silver nitrate (AgNO_3_) as a precursor and 10% *M. indica* aqueous leaf extract as both reducing and capping agent [14]. Leaf extract and AgNO_3_ (0.02 M) solutions were mixed in the ratio of 9:1 and kept for reaction in the dark for approximately 24 h. The plant extract reduced the silver nitrate and led to the formation of AgNPs. The change in solution color from brown to blackish indicated the formation of AgNPs. The reduction process was monitored by using UV-Visible spectroscopy.

### 2.4. Characterization of Green Synthesized Silver Nanoparticles (AgNPs)

The synthesized nanoparticles were characterized in terms of shape, size, and morphology using UV-Visible spectrophotometry (UV-Vis), particles size analyzer (PSA), scanning electron microscope (SEM), and X-ray diffraction (XRD). Spectral analysis of AgNPs was performed using an ELICO UV1900, Hyderabad, India, Double Beam Spectrophotometer with spectrum scanning in the wavelength range of 190–1100 nm with a band width of 2 nm. Green nanoparticles were analyzed at a wavelength ranging from 200 to 700 nm. Characterization of AgNPs in terms of mean diameter and distribution was performed using PSA (Nicomp, NANOZ Z3000 PSS, Billerica, MA, USA). To assess the topology of green synthesized nanoparticles, SEM (Carl Zeiss-EVO-18- Cambridge, UK) was used. An X-ray Diffractometer (Powder) (Model: SmartLab SE, Tokyo, Japan) with fully automated alignment under computer control SAXS capabilities, with Optional D/teX Ultra-high-speed, position-sensitive detector system with 2D: 2–150°, was used to understand the structural information of AgNPs.

### 2.5. Biochemical Analysis

The seeds were procured from the Durum Wheat Main Agricultural Research Station of the AICRP (All India Coordinated Research Projects), at the University of Agricultural Sciences in Dharwad. The tests were performed in a factorial complete-replicate design with three replicates. The variety of durum wheat employed in this study was Bijaga Yellow which is a tall durum wheat variety cultivated in rain fed regions.

Wheat plant was evaluated by pot culture experiments in a polyhouse. Blast disease was inoculated artificially after 40 days of sowing. The AgNPs treatment was given after the development of disease as described in the study design (Table 1). Leaves samples were collected after 2–3 days of treatment for determining the reducing sugar and total phenol and antioxidant enzyme activities, such as superoxide dismutase (SOD), catalase (CAT), glutathione reductase (GR), peroxidase (POX), and phenylalanine ammonia lyase (PAL).

### 2.6. Extraction of Enzyme and Determination of Protein Content

In the present work, enzymes were extracted from freshly collected leaf tissues at 0–4 °C. The antioxidant enzyme activities of SOD, CAT, POX, GR, and PAL were determined by extracting 0.5 g of leaf tissues with 2 mL of respective buffers (0.05 M sodium phosphate buffer of pH 7.8 for SOD, pH 7.0 for CAT and POX, 0.1 M Tris-HCl buffer of pH 7.8 containing 2 mM dithiothreitol for GR and 0.1 M Tris buffer of pH 8.5 for PAL). The homogenates were centrifuged at 14,000 rpm for 20-min at 4 °C. The resulting supernatant was used as an enzyme source for determining antioxidant activities and the protein content was measured using Lowry’s method.

### 2.7. Antioxidant Enzyme Assays

#### 2.7.1. Catalase

CAT (EC 1.11.1.6) activity of Wheat leaves was analyzed using the Beers and Sizers spectrophotometric technique [15]. To commence the reaction, 20 µL of enzyme extract was added to a reaction mixture containing 2.98 mL of 16.65 mM hydrogen peroxide in 50 mM phosphate buffer, pH 7.0. The decrease in absorbance was monitored at 240 nm against substrate blank for a period of 3 min. Specific activity was expressed as µM min^−1^ mg^−1^ protein and one unit of CAT was defined as 1 µM of H_2_O_2_ degraded per minute at pH 7.0 and 25 °C.

#### 2.7.2. Superoxide Dismutase

Using the Beauchamp and Fridovich method [16], the SOD activity in wheat leaves was measured photochemically at 560 nm. The composition of 3 mL of reaction mixture included 50 mM phosphate buffer (pH 7.8), 20 µL enzyme extract, 10 mM L-methionine, 33 µM p-nitroblue tetrazolium chloride (NBT), 0.66 µM ethylene diamine tetra acetic acid (EDTA), and 3.3 µM riboflavin. The addition of riboflavin was immediately followed by illuminating the glass tube with the light of a 15 W fluorescent lamp at 25 °C for 20 min. Absorbance at 560 nm was read to determine the concentration of blue formazan formed by NBT photoreduction against a blank which comprised of same reaction mixture excluding the exposure to light. The specific activity of SOD is measured in international units (IU) per milligram of protein, and one unit of SOD is defined as the quantity of enzyme needed to inhibit 50% of the NBT photo-reduction per minute.

#### 2.7.3. Peroxidase Activity

Peroxidase activity (POX, EC 1.11.1.7) in wheat leaves was measured kinetically by the method of Chance and Maehly [17]. The addition of 20 µL of the enzyme extract to reaction mixture, containing 2.88 mL of 0.1 M potassium phosphate buffer (pH 7.0), 50 µL of 0.02 M guaiacol, and 50 µL of 0.042% hydrogen peroxide, initiated the reaction and the increase in optical density at 436 nm was recorded for 5 min. The specific activity of POX was quantified as µM of oxidized guaiacol produced per minute per milligram of protein.

#### 2.7.4. Glutathione Reductase

The spectrophotometric quantification of GR (EC 1.8.1.7) activity in wheat leaves was performed following the method of Mavis and Stellwagen [18]. Composition of reaction mixture included 0.1 mL of 30 mM oxidized glutathione, 1.5 mL of 100 mM potassium phosphate buffer with 3.4 mM EDTA, pH 7.6, 0.35 mL of 0.8 mM reduced ß-nicotinamide adenine dinucleotide phosphate (NADPH), and 0.95 mL of distilled water. The addition of 100 µL of enzyme extract commenced the reaction and the reduction in absorbance at 340 nm was monitored for 5 min. GR specific activity is represented as µM min^−1^ mg^−1^ protein, and one GR unit is equal to the quantity of enzyme required to oxidize 1 µM of NADPH per minute at pH 7.6 and 25 °C.

#### 2.7.5. Phenylalanine Ammonia Lyase

Using the spectrophotometric approach developed by Paltonen and Karjalainen [12], the PAL (EC.4.3.1.5.) activity in wheat leaves was measured. The assay mixture containing 0.5 mL of enzyme extract and 2.5 mL of 0.2% L-phenylalanine in 0.1 mL of Tris buffer, pH 8.5 was incubated for 1-h at 40 °C and the reaction was terminated with 0.2 M HCl. The absorbance was measured at 290 nm against a substrate blank. The specific activity of PAL was reported in units of µM of trans-cinnamate released per minute per milligram of protein. One unit of PAL is defined as the quantity of enzyme necessary to liberate 1 µM of trans-cinnamate from L-phenylalanine per minute.

### 2.8. Estimation of Reducing the Sugar

For the quantitative analysis of reducing sugars in wheat leaves, 1 g of dried leaf material was introduced in hot ethanol (80%) for 15 min and then homogenized with pestle and mortar. The homogenate thus obtained was filtered using muslin cloth, the residue was re-extracted two or three times, and the filtrate were made up to final volume of 10 mL with 80% ethanol. Reducing sugars were quantified by employing Nelson Somogi’s method (Norton Nelson, 1944) in which the reducing sugars in alkaline medium reduce cupric ions to cuprous ions which form a chromophore with arsenomolybdate reagent and have a λmax at 510 nm. The reducing sugar was expressed as mg per gram dry weight.

### 2.9. Estimation of Total Phenols

The Folin-Ciocalteau reagent (FCR) method was used to estimate the total phenols present in wheat leaf samples. The reaction between phenols and the oxidizing agent phosphomolybdo-phosphotungstate in FC reagent results in the formation of blue-colored chromophore with a maximum absorption at 660 nm. The amount of phenol has a direct correlation with the development of color with the reagent. Total phenols were extracted using 10.0 mL of hot 80% alcohol with 1 g of dried leaf as the starting material (Sadasivam and Manikam, 1992). The colorimetric method of (Bray and Thorpe, 1954.) was used for the determination of total phenols using the Folin–Ciocalteau reagent. The phenol content was expressed as mg per gram dry weight.

### 2.10. Bacterial Strains Used in This Study

We used 15 multidrug-resistant (MDR) bacteria comprising gram negative bacteria-*Escherichia coli* (*n* = 6), *Klebsiella pneumoniae* (*n* = 7) and gram-positive bacteria- Methicillin Resistant *Staphylococcus aureus* (*n* = 2). These clinical strains were phenotypically and genotypically characterized in our previous study and found to possess wide spectrum of beta lactamase genes responsible for antibiotic resistance [17]. ATCC 25922 *E. coli* was used as a control strain.

### 2.11. In Vitro Antibiotic Susceptibility Test of M. indica Nano Particles (MNPs) against MDR Bacteria

#### Disc Diffusion Method

The antibacterial activity of MNPs against the selected MDR bacterial (*n* = 15) strains was carried out using the Kirby–Bauer disc diffusion method [18]. The bacterial strains were grown until attaining McFarland standard O.D of 0.5. The inoculum was spread in Muller Hinton Agar (MHA) (Himedia, India) using sterile cotton swabs and sterile antimicrobial susceptibility disks were loaded on the plates with 10 µL of MNP. The plates were incubated at 37 °C for 48 h and zone of inhibition was recorded.

### 2.12. Statistical Analysis

The data of the experiment were analyzed statistically by the procedure described by Gomez and Gomez [19].

## 3. Results and Discussion

### 3.1. Characterization of Green Synthesized AgNPs

#### 3.1.1. The UV-Visible Absorption Spectrum of Green Synthesized AgNPs

**Figure 1:**
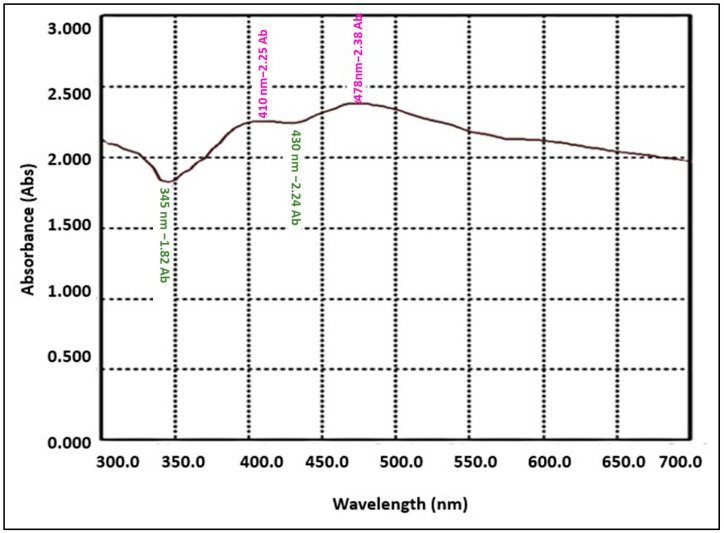
UV–Visible absorption spectrum of green synthesized AgNPs using *M. indica* aqueous leaf extract.

The addition of *M. indica* leaf extract to the silver nitrate solution led to the change in color from brown to dark blackish-brown, indicating the formation of AgNPs due to the reduction of silver ions by the reducing agents present in the *M. indica* leaf extract. Further confirmation of AgNPs formation was performed using the UV- visible spectrophotometer. Surface plasmonic resonance absorbance range between 410 and 480 nm was, in particular, used as an indicator to confirm the reduction of Ag+ to metallic Ag^o^ [20]. The strong and broad surface plasmonic resonance (SPR) peak centered at 475 nm and 410 nm confirmed the formation of AgNPs (Figure 1).

#### 3.1.2. AgNPs Particle Size Distribution Study by Particle Size Analyzer (PSA)

**Figure 2:**
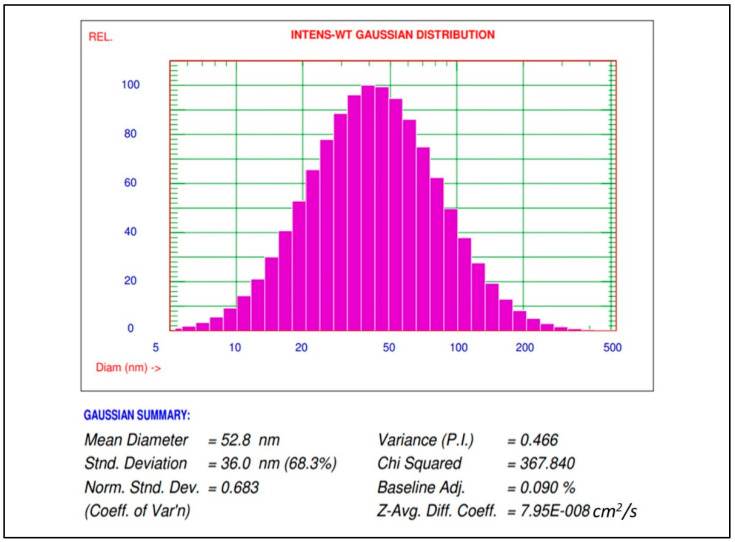
Particle size distribution of AgNPs synthesized using *M. indica* aqueous leaf extract.

Nanoparticles size distribution and mean diameter of green synthesized AgNPs using *M. indica* aqueous leaf extract were characterized using PSA (Nicomp NANOZ Z3000 PSS). PSA showed a mean diameter at 52.8 nm and nanoparticle size distribution from 5 nm to 220 nm (Figure 2).

#### 3.1.3. SEM Micrographs of Green Synthesized AgNPs

**Figure 3:**
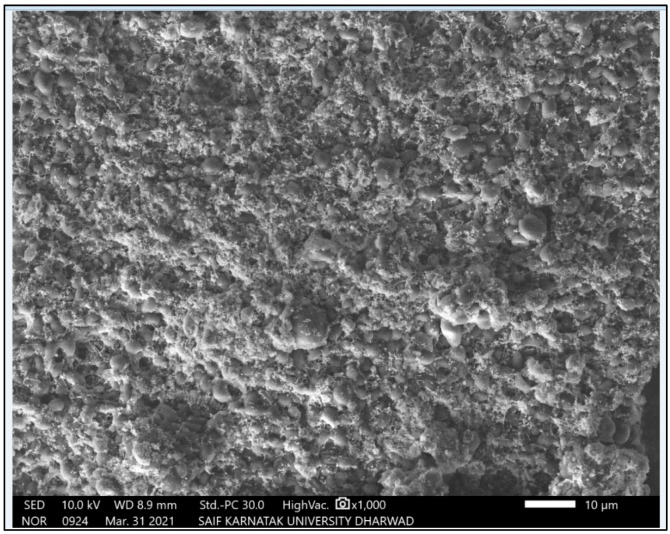
SEM micrograph of green synthesized AgNPs using *M. indica* aqueous leaf extract.

Scanning electron microscopy imaging was carried out to view the morphology and size of the silver nanoparticles. SEM micrograph obtained for the AgNPs synthesized using *M. indica* leaf extract showed high-density nanoscale particles (52 nm) with spherical shape (Figure 3). SEM is a surface imaging technique that can resolve micro- and nano-scale differences in particle size, particle size distribution, nanomaterial type, and particle surface morphology. Through scanning electron microscopy (SEM), we can analyze particle morphology and create a histogram from images, either manually by measuring and counting the particles or automatically using specialized software. Our findings are in line with previous studies involving nanoparticles synthesized using *Raphanus sativus* and *Vitex leucoxylon* extract [21,22].

#### 3.1.4. XRD Spectrum of AgNPs Synthesized by Using *M. indica* Leaf Extract

**Figure 4:**
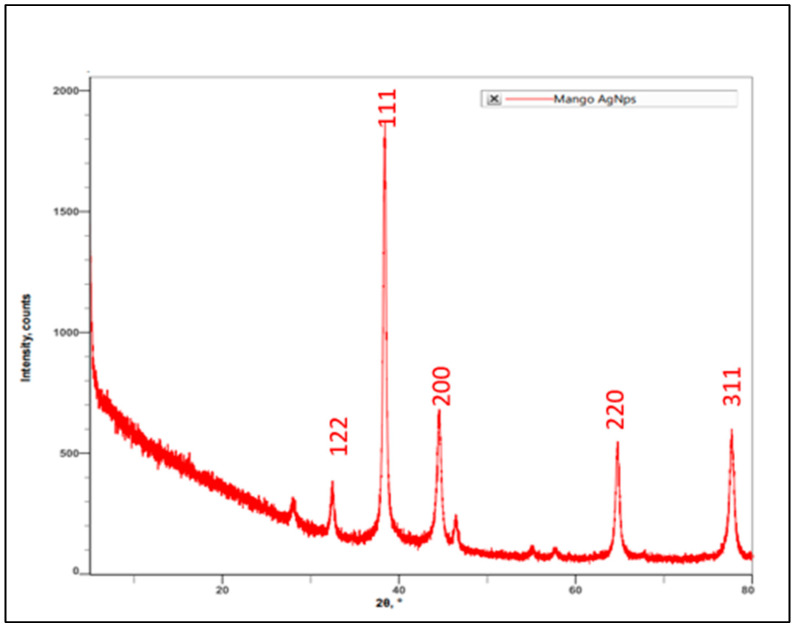
XRD graph of green synthesized AgNPs using *M. indica aqu*eous leaf extract.

Characterization of AgNPs synthesized by using *M. indica* leaf extract was carried out by performing XRD spectrum analysis. The result indicated peaks angle of 2θ at 27.98°, 32.42°, 38.31°, 44.46°, 46.34°, 54.93°, 57.61°, 64.63°, and 77.53°. The highest intensity counts were at the angle of 38.31°, followed by 44.46°, 77.58°, 64.63°, 32.42°, 46.34°, and 27.98° for leaf extracts which can be assigned the plane of silver crystals (Figure 4). When compared with the standard (JCPDS No. 87-0579), the obtained XRD spectrum confirmed that the synthesized silver nanoparticles were in nanocrystal form and crystalline in nature. The peaks can be assigned to the planes (122), (111), (200), (220), and (311) facet of silver crystal, respectively. These results corroborate the findings of earlier studies [23,24].

### 3.2. Estimation of Primary and Secondary Metabolites in AgNPs Treated Wheat Plants Infected with Spot Blotch Infection (Bipolaris sorokiniana)

#### 3.2.1. Reducing Sugar

Levels of reducing sugar in the AgNPs -treated plants showed a slight increase when compared to the diseased wheat plant (2.73 g % dry weight) (Table 2). Among the treatments, plants treated with a higher concentration of silver nanomaterials (30 ppm: 6.94 g % and 50 ppm: 7.82 g % dry weight) showed a phenomenal increase in reducing sugar content compared to plants treated with lower concentration (5 ppm: 4.44 g %, 10 ppm: 5.00 g %, 15 ppm: 5.45 g %, and 20 ppm: 5.87 g % dry weight) and diseased plant (2.34 g % dry weight). In comparison to control, the disease inoculated wheat plant leaves recorded a reduction in reducing sugar content (2.73 g % dry weight) as compared to control leaves (10.61 g % dry weight). 

#### 3.2.2. Total Phenol

Total phenol content in all nano-treated plants showed a slight increase when compared to the diseased wheat plants (1.65 g % dry weight). Among the treatments, plants treated with a higher concentration of AgNPs (30 ppm: 1.98 g %, 50 ppm: 2.09 g % dry weight) showed a phenomenal increase in total phenol content compared to low concentration (5 ppm: 1.72 g %, 10 ppm: 1.76 g %, 15 ppm: 1.81 g %, and 20 ppm: 1.85 g % dry weight) and diseased plant (1.65 g % dry weight). In comparison to control, the disease inoculated wheat plant leaves recorded a higher total phenol content (1.65 g % dry weight) as compared to control leaves (1.2 g % dry weight) (Table 3).

### 3.3. Estimation of Stress Response Enzyme Activities in AgNPs Treated Wheat Plants Infected with Bipolaris sorokiniana

#### 3.3.1. Estimation of SOD Activity

SOD activity increased by nearly 35.53% in the diseased plants compared to the control plants. All AgNPs treated plants showed elevation in SOD activity in all different treatments (30 ppm: 3.91 U/mg protein and 50 ppm: 4.42 U/mg protein). The AgNPs treated plants showed significant elevation in enzyme activity and a reduction in disease severity. In T2, T3, T4, and T5 treatments, crops showed elevation in enzyme activity but no significant reduction in disease severity. At 5 ppm: 38.98%, 10 ppm: 38.80%, 15 ppm: 42.41%, 20 ppm: 42.41% elevation in enzyme activity was recorded (Table 4).

#### 3.3.2. Estimation of CAT Activity

CAT activity increased by nearly 22.22% in the diseased plants compared to the control plants. All AgNPs treated plants showed elevation in CAT activity in all different treatments. At 30 ppm: 492 U/mg protein and 50 ppm: 583 U/mg protein, the AgNPs treated plants showed significant elevation in enzyme activity and a reduction in disease severity. In T2, T3, T4, and T5, crops showed elevation in enzyme activity but no significant reduction in disease severity. At 5 ppm: 24.49%, 10 ppm: 22.76%, 15 ppm: 30.29%, and 20 ppm: 28.05% elevation in enzyme activity was recorded (Table 5).

#### 3.3.3. Estimation of POX Activity

POX activity increased by nearly 40.61% in the diseased plants compared to the control plants. All AgNPs treated plants showed elevation in POX activity in all different treatments. At 30 ppm: 3.00 U/mg protein (61.00%) and 50 ppm: 3.24 U/mg protein (63.89%), the nanoparticle treated plants showed significant elevation in enzyme activity and a reduction in disease severity. In T2, T3, T4, and T5, plants showed elevation in enzyme activity but no significant reduction in disease severity. At 5 ppm: 52.24%, 10 ppm: 56.5%, 15 ppm: 57.30%, and 20 ppm: 59.52% elevation in enzyme activity was recorded (Table 6).

#### 3.3.4. Estimation of GR Activity

GR activity increased by nearly 39.37% in the diseased plant compared to the control plant. All AgNPs treated plants showed elevation in GR activity in all different treatments. At 30 ppm: 3.79 U/mg protein and 50 ppm: 4.00 U/mg protein, the nanoparticle treated plant showed significant elevation in enzyme activity and a reduction in disease severity. In T2, T3, T4, and T5, wheat plants showed elevation in enzyme activity but no significant reduction in disease severity. At 5 ppm: 49.00%, 10%: 49.50%, 15 ppm: 53.0%, and 20 ppm: 54.70% elevation in enzyme activity was recorded (Table 7).

#### 3.3.5. Estimation of PAL Activity

PAL activity increased by nearly 32.98% in the diseased plant than in the control plant. All AgNPs treated plants showed elevation in PAL activity in all different treatments. At 30 ppm: 6.36 U/mg protein and 50 ppm: 6.78 U/mg protein, the nanoparticle treated plants showed significant elevation in enzyme activity and a reduction in disease severity. In T2, T3, T4, and T5, crops showed elevation in enzyme activity but no significant reduction in disease severity. At 5 ppm: 34.92%, 10 ppm: 34.92%, 15 ppm: 33.45%, and 20 ppm: 39.37% elevation in enzyme activity was recorded (Table 8).

### 3.4. Antibacterial Activity against MDRs

**Figure 5:**
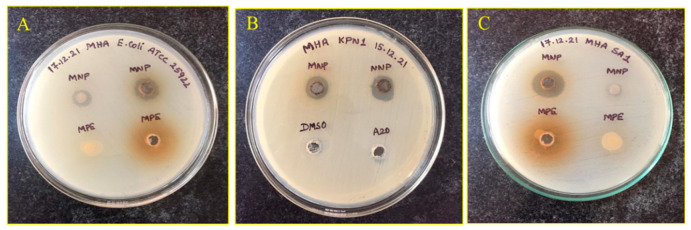
Zone of Inhibition (visible clear zones) produced by MNP against clinical MDR pathogens: (**A**): *E. coli* ATCC 25922 (**B**) *K. pneumoniae* KP1 and (**C**): Methicillin Resistant *S. aureus* (MRSA-SA1).

The AgNPs showed antibacterial activity against all the MDR pathogens included in this study. The results of disc diffusion assay are summarized in Table 9. The clearly visible zone of inhibition exhibited by MNP against the Gram-negative and -positive MDR pathogens is depicted in Figure 5. The scientific name of the bacterial strain used in the study is presented in Table 10.

### 3.5. Green Synthesis of AgNPs by Using M. indica Leaf Extract and Their Characterization

The present study reports the successful synthesis and characterization of green synthesized AgNPs using *M. indica* aqueous leaf extract. Green synthesis of AgNPs using different plant sources has been well documented in the literature [14,25]. In the present study, we chose *M. indica* leaf extract for the green synthesis of AgNPs because it has a significant amount of reducing activity and capping properties due to the presence of phytochemicals, e.g., alkaloids, phenols, flavonoids, alkaloids, fats, amino acids, saponins, and oils. These bioactive compounds efficiently reduce silver salts (Ag^+^) to Ag^0^ [26]. Another reason for *M. indica* leaf selection is that some of the phytochemicals present in the plant extracts exhibit antimicrobial properties. In addition, *M. indica* leaf extract*s* not only efficiently synthesize silver nanoparticles, but also do so through capping.

Green synthesized AgNPs were characterized for their mean size, distribution, shape, morphology, and topography of nanoparticles. In this study, phytosynthesized AgNPs were initially identified by the change in color from light brown to blackish, indicating the formation of AgNPs. The present results were reported from colorless to brown grey in previous studies [27,28].

Further confirmation of AgNPs formation was ascertained by a surface plasmon resonance peak centered at 475 nm with a median nanoparticle size of 52 nm. A similar AgNPs size was observed in other studies. For instance, *M. indica* leaf extract based AgNPs size was reported to be 20–55 nm with plasmon resonance peak at 452 nm, 5–40 nm with plasmon resonance peak at 470 nm [29], 10–26 nm with surface plasmon resonance peak at 450 nm [30], and 32 nm with a surface plasmon peak at 393 nm [31]. *Catharanthus roseus* based silver nanoparticles were evaluated as 20–50 nm with a peak absorbance at 500 nm [27], 49 nm with surface plasmon resonance absorption peak at 425 nm [32], and 40–60 nm [33]. AgNPs other than the spherical shape can also contribute to the shift in surface plasmon resonance peak towards 500 nm and above.

SEM and XRD results confirmed the presence of AgNPs. SEM micrographs showed spherical shape AgNPs and the presence of crystalline AgNPs through the XRD spectrum analysis [34,35]. The SEM micrograph revealed phytosynthesized AgNPs using *Catharanthus roseus* leaf extract present as a bunch form in shape [33].

### 3.6. Studies on Biochemical Changes in Wheat Plants Infected with Spot Blotch (Bipolaris sorokiniana)

#### 3.6.1. Reducing Sugars

Sugars constitute the primary biomolecule providing energy and structural material for defense responses in plants, and also act as signal molecules interacting with the plant immune system. Sugars cause oxidative burst at the early stages of infection, increasing the lignification of cell walls, stimulating the synthesis of flavonoids, and inducing certain PR proteins. Some sugars act as priming agents, inducing higher plant resistance to pathogens reported [36].

In the present study, the response of AgNPs applied to wheat crops infected with spot blotch was studied to correlate the role of reducing sugar content upon spot blotch infection and nanoparticle application. As per the results obtained (Table 1), the control plant recorded the highest reducing sugar content, and the lowest reducing sugar content was observed in the spot blotch infected plant(T_1_). Spot blotch induced a decrease in sugar content. All other treatments showed elevation in reducing sugar content after the application of silver nanoparticles.

Carbohydrates and proteins are the primary metabolites that are exploited by biotic stress inducers for their growth and development. These primary metabolites also function as a precursor for secondary substances, which play a major role in plant defense [37]. Alberto (2014) [38] observed a decrease in reducing sugar content in the disease-infected plant compared to the healthy plants in their study on biochemical changes in *Theiumcepa L*. infected with anthracnose. Kumar et al. (2011) [39] reported that reducing sugar was decreased after infection of spot blotch infection in barley. Yanik F and Vardar F (2019) [40] observed the elevation in reducing sugar content in wheat plants applied with silver nanoparticles at different concentrations. Krishnaraj et al. (2012) in their study on biologically synthesized AgNPs found that these particles exerted a slight stress condition on the growth and metabolism of *B. monnieri* [41] accompanied by an elevation in total sugar content based on concentration applied.

#### 3.6.2. Total Phenol

Phenols play an important role in the plant defense system against plant pathogens, insects, and the cyclic reduction of reactive oxygen species (ROS), such as superoxide anion and hydroxide radicals, H_2_O_2_, and singlet oxygen [42].

In the present study, the response of AgNPs applied to wheat plants infected with spot blotch was studied to correlate the role of total phenol content upon spot blotch infection and nanoparticle application. As per the results obtained (Table 2) among control and disease infected wheat crops, the healthy plant showed a low amount of total phenol. AgNPs treated spot blotch infected wheat plants showed elevation in total phenol content correlating the concentration of nanoparticles applied, and reduction in severity was also observed at higher concentration nanoparticle application.

Similar results were reported earlier whereby rust-resistant sorghum genotypes recorded higher phenols as the age of the crop increased [43]. A similar study on spot blotch (*Bipolaris sorokiniana*) in healthy and infected leaves of barley (*Hordeum vulgare*) indicated that resistant genotypes had more amount of total phenols content than susceptible genotypes [44]. Phenols inhibit disease development through different mechanisms like inhibition of extracellular fungal enzymes (cellulase, pectinase, laccase and xylanase), fungal oxidative phosphorylation, nutrition deprivation and antioxidant activity in plant tissue [45]. A similar study on the effect of nanoparticle application on spot blotch infected barley biochemical systems also showed an elevation in total phenol content associated to nanoparticle application concentration [39].

#### 3.6.3. Superoxide Dismutase

SOD catalyzes the dismutation of superoxide radicals to hydrogen peroxide and oxygen, and constitutes the most important enzyme in cellular defense, because its activation directly modulates the amount of superoxide anion^-^ and H_2_O_2_ [46]. SOD activity may reduce the risk of hydroxyl radical formation. SODs are classified into three major groups based on their metal cofactor, and they are iron SOD (Fe-SOD) and copper-zinc SOD (Cu/Zn-SOD) are localized in the chloroplast, cytosol; manganese SOD (Mn-SOD) is found mainly in mitochondria and peroxisomes [47].

In the present study, biochemical changes in AgNPs treated wheat plants infected with spot blotch disease were analyzed. The wheat plants showed elevation in the SOD activity in all silver nanoparticles treated conditions when compared with the control. As per the results obtained (Table 4), all the AgNPs treated wheat plants showed an elevation in SOD activity, whereas only the 30 ppm and 50 ppm silver AgNPs treatment plants showed a reduction in spot blotch severity and elevation in SOD activity.

Similar results were observed [41] in studies on the effect of AgNPs application on *B. monnieri* biochemical activity and the treated plants showed an elevation in SOD activity at 100 ppm AgNPs treatment. Similar results were observed by Du et al. (2015), who studied the effect of AgNPs application on wheat plants and recorded an elevation in SOD activity at 100 mg/kg nanoparticle application [48].

#### 3.6.4. Catalase

Plants possess several antioxidant enzymes that eliminate reactive oxygen species (ROS). CAT enzymes have a defensive role against ROS. They are responsible for the removal of toxic H_2_O_2_ in the cells, thereby protecting the cells from getting damaged. Up to a certain level, ROS production under stress may work as a signal for triggering defense responses via transduction pathways [49,50]. A high amount of ROS production in the cell causes cell death.

In the present study, the wheat plants showed elevation in the catalase activity in all AgNPs treated conditions when compared with a control. As per the results obtained (Table 4), all the silver nanoparticle treated wheat plants showed elevation in CAT activity but there was no reduction in disease severity in other treatments, except at 30 ppm and 50 ppm AgNPs treatment, where the infected plants showed a reduction in spot blotch severity and elevation in catalase activity.

Similar results were obtained in the study on AgNPs induced morphological, physiological, and biochemical changes in the wheat plant. Elevation in all of the antioxidative enzymes and phenol content was recorded [51]. A similar study conducted by Mohamed et al., in 2017, which also showed corresponding biochemical changes in a crop treated with nanoparticles [51,52].

#### 3.6.5. Peroxidase (POX)

A heme-containing protein called peroxidase (POX) preferentially oxidizes aromatic electron donors at the expense of H_2_O_2_. By using oxidative polymerization, POX facilitates the conversion of cinnamyl alcohol to lignin. Vascular plants have many POX genes, and each class of these genes may have a unique physiological function in preventing oxidative damage to the cell membrane [53].

In the present study, the wheat plants showed elevation in the peroxidase activity in all of the AgNPs treated conditions when compared with a control. As per the results obtained (Table 5), all the AgNPs treated wheat plants showed elevation in POX activity, but there was no reduction in disease severity in other treatments except at 30 ppm and 50 ppm AgNPs treatment. With 30 ppm and 50 ppm silver nanoparticle treatment, crops showed a reduction in spot blotch severity and elevation in peroxidase activity was recorded. The present study indicates the crop showed more resistance towards disease at 30 and 50 ppm silver nanoparticles, but all treatments showed elevation in all plant biochemical constituents.

Numerous fungal species, including *Trichosporium vesiculosum*, *Macrophomin phaseolina*, *Coprinus comatus*, *Mycophaerella arachidicola*, *Fusarium exosporium*, and *Botrytis cenere*, have been shown to be resistant to POX in some studies [53,54]. Following inoculation with stem and leaf rust pathogens, low infection type isogenic lines of wheat showed a quicker increase in POX activity than sensitive isogenic lines of wheat [55].

#### 3.6.6. Glutathione Reductase

Another important pathway involved in the control of ROS levels in the plant tissue is the ascorbate/glutathione cycle in which Glutathione reductase plays a major role [56]. GR is a flavoprotein oxidoreductase that catalyzes the reduction of glutathione disulphide to the sulfhydryl form GSH. This enzyme employs NADPH as a reductant.

As per the results obtained (Table 6), all treatments showed elevation in GR activity, but a reduction in disease severity was observed in only 30 ppm and 50 ppm AgNPs treatment conditions. In the present study, the wheat plant showed elevation in the GR activity in all AgNPs treated conditions when compared with the control.

Similarly, a rapid elevation in GR content was observed by Yanik F and Vardar F, 2019, in their study on silver nanoparticle-induced morphological, physiological, and biochemical changes in the wheat plant [40]. An elevation in all antioxidant enzyme and phenol content was recorded.

#### 3.6.7. Phenylalanine Ammonia-Lyase

The primary enzyme in the plant phenylpropanoid pathway, phenylalanine ammonia-lyase catalyzes the synthesis of secondary metabolites from L-phenylalanine, such as lignin, flavonoids, and phytoalexins. L-phenylalanine is non-oxidatively deaminated by the PAL enzyme, resulting in the formation of trans-cinnamic acid and a free ammonium ion. Phenylalanine, an amino acid, is converted to trans-cinnamic acid in plants, which is the first step in the transfer of carbon from primary metabolism to phenylpropanoid secondary metabolism.

As per the results obtained (Table 7) among all the treatments, T6 and T7 recorded the highest PAL activity, whereas the lowest PAL activity was observed in the control plant. Spot blotch infected plants also showed a small elevation in PAL activity. In the present study, biochemical changes in AgNPs treated wheat crops infected with spot blotch disease were analyzed. The wheat plant showed elevation in the peroxidase activity in all AgNPs treated conditions when compared with the control condition. As per the results obtained, all the AgNPs treated wheat plants showed elevation in PAL, but no reduction in disease severity was observed. With 30 ppm and 50 ppm silver nanoparticle treatment, crops showed a reduction in spot blotch severity and elevation in PAL activity was recorded. The present study indicates the crop showed more resistance towards disease at 30 ppm and 50 ppm, but all treatments showed elevation in all plant biochemical constituents.

It is possible that the rapid induction of PAL genes in resistant interactions between groundnut and its pathogen/elicitor is caused by the involvement of a signal transduction mechanism. This mechanism is triggered specifically as a result of the interaction between elicitor and receptor molecules, thereby showing differential transcriptional rates of PAL compatible and incompatible interactions. A mechanism quite similar to this one was postulated earlier in several plants [56,57]

## 4. Conclusions

Silver nanoparticles were successfully synthesized by using *Mangifera indica* leaf extract with a size below 100 nm. The formation of AgNPs was confirmed using various analytical techniques, such as UV-visible spectrophotometer, particle size analyzer (PSA), scanning electron microscopy (SEM), and X-ray diffraction. Biochemical analysis of silver nanoparticle treated wheat crop infected with spot blotch showed elevation in all biochemical constituents, e.g., reducing sugar, total phenol, superoxide dismutase (SOD), catalase (CAT), peroxidase (POX), glutathione reductase (GR), and phenylalanine ammonia-lyase (PAL). Elevation in all biochemical constituents was recorded under all treatment conditions but a reduction in disease severity was recorded only in 30 ppm and 50 ppm concentration AgNPs treatments. The overall study revealed that the higher amount of reducing sugar, total phenols, antioxidant enzyme activity, and phytochemical precursor enzyme PAL plays an important role in the defense mechanism of plants against wheat spot blotch infection. Moreover, this study revealed that silver nanoparticles application on wheat crops causes the elevation in antioxidative enzyme activity and the amount of total phenol, reducing sugar content, and promising antibacterial activity against MDR strains.

## Figures and Tables

**Table 1:** Details of the treatment groups used in the study.

Variety of Durum Wheat	Treatment	Replication
Bijaga Yellow	C: Control leaves T_1_: Diseased + 0 ppm AgNPs (Disease control) T_2_: Diseased + 5 ppm AgNPs T_3_: Diseased + 10 ppm AgNPs T_4_: Diseased + 15 ppm AgNPs T_5_: Diseased + 20 ppm AgNPs T_6_: Diseased + 30 ppm AgNPs T_7_: Diseased + 50 ppm AgNPs	Three

**Table 2:** Estimation of reducing sugar in spot blotch infected wheat plant leaves in response to nanoparticle treatment.

Treatments	Reducing Sugar	
	g % Dry Weight	% Variation
Control	10.61	-
T_1_: 0 ppm AgNPs	2.73	−288.645
T_2_: 5 ppm AgNPs	4.44	−138.964
T_3_: 10 ppm AgNPs	5.00	−112.20
T_4_: 15 ppm AgNPs	5.45	−94.67
T_5_: 20 ppm AgNPs	5.87	−80.74
T_6_: 30 ppm AgNPs	6.94	−52.88
T_7_: 50 ppm AgNPs	7.82	−35.67
SE (m)	0.22	
C.D.	0.67	

**Table 3:** Estimation of total phenol in spot blotch infected wheat plant leaves in response to nanoparticle treatment.

Treatments	Total Phenol	
	g % Dry Weight	% Variation
Control	1.2	-
T_1_: 0 ppm AgNPs	1.65	27.27
T_2_: 5 ppm AgNPs	1.72	30.23
T_3_: 10 ppm AgNPs	1.76	31.81
T_4_: 15 ppm AgNPs	1.81	33.70
T_5_: 20 ppm AgNPs	1.85	35.13
T_6_: 30 ppm AgNPs	1.98	39.39
T_7_: 50 ppm AgNPs	2.09	42.58
SE (m)	0.05	
C.D.	0.16	

**Table 4:** Estimation of Superoxide dismutase activity in spot blotch infected wheat plant leaves in response to nanoparticle treatment.

Treatments	Superoxide Dismutase	
	U/mg Protein	% Variation
Control	2.05	-
T_1_: 0 ppm AgNPs	3.18	35.53
T_2_: 5 ppm AgNPs	3.36	38.98
T_3_: 10 ppm AgNPs	3.35	38.80
T_4_: 15 ppm AgNPs	3.54	42.09
T_5_: 20 ppm AgNPs	3.56	42.41
T_6_: 30 ppm AgNPs	3.91	47.57
T_7_: 50 ppm AgNPs	4.42	53.61
SE (m)	0.11	
C.D.	0.34	

**Table 5:** Estimation of catalase activity in spot blotch infected wheat plant leaves in response to nanoparticle treatment.

Treatments	Catalase	
	U/mg Protein	% Variation
Control	336	-
T_1_: 0 ppm AgNPs	432	22.22
T_2_: 5 ppm AgNPs	445	24.49
T_3_: 10 ppm AgNPs	435	22.76
T_4_: 15 ppm AgNPs	482	30.29
T_5_: 20 ppm AgNPs	461	28.05
T_6_: 30 ppm AgNPs	492	31.71
T_7_: 50 ppm AgNPs	583	42.37
SE (m)	14.77	
C.D.	44.68	

**Table 6:** Estimation of Peroxidase activity in spot blotch infected wheat plant leaves in response to nanoparticle treatment.

Treatments	Peroxidase	
	U/mg Protein	% Variation
Control	1.17	-
T_1_: 0 ppm AgNPs	1.97	40.61
T_2_: 5 ppm AgNPs	2.45	52.24
T_3_: 10 ppm AgNPs	2.69	56.51
T_4_: 15 ppm AgNPs	2.74	57.30
T_5_: 20 ppm AgNPs	2.89	59.52
T_6_: 30 ppm AgNPs	3.00	61.00
T_7_: 50 ppm AgNPs	3.24	63.89
SE (m)	0.07	
C.D.	0.22	

**Table 7:** Estimation of Glutathione reductase activity in spot blotch infected wheat plant leaves in response to nanoparticle treatment.

Treatments	Glutathione Reductase	
	U/mg Protein	% Variation
Control	1.54	-
T_1_: 0 ppm AgNPs	2.54	39.37
T_2_: 5 ppm AgNPs	3.02	49.00
T_3_: 10 ppm AgNPs	3.05	49.50
T_4_: 15 ppm AgNPs	3.28	53.04
T_5_: 20 ppm AgNPs	3.40	54.70
T_6_: 30 ppm AgNPs	3.79	59.36
T_7_: 50 ppm AgNPs	4.00	61.50
SE (m)	0.12	
C.D.	0.38	

**Table 8:** Estimation of Phenylalanine ammonia lyase activity in spot blotch infected wheat plant leaves in response to nanoparticle treatment.

Treatments	Phenylalanine Ammonia Lyase	
	U/mg Protein	% Variation
Control	3.82	-
T_1_: 0 ppm AgNPs	5.70	32.98
T_2_: 5 ppm AgNPs	5.87	34.92
T_3_: 10 ppm AgNPs	5.74	33.45
T_4_: 15 ppm AgNPs	6.13	37.68
T_5_: 20 ppm AgNPs	6.20	39.37
T_6_: 30 ppm AgNPs	6.36	39.94
T_7_: 50 ppm AgNPs	6.78	43.66
SE (m)	0.17	
C.D.	0.51	

**Table 9:** Diameter of zone of inhibition (mm) observed for MNP against MDR pathogens.

	Bacteria	Diameter of Inhibition Zone (mm)
		MNP
1.	*ATCC 25922 E. coli*	12
2.	*E. coli Ec1*	11
3.	*E. coli Ec2*	13
4.	*E. coli Ec3*	11
5.	*E. coli Ec4*	12
6.	*E. coli Ec5*	12
7.	*E. coli Ec6*	11
8.	*K. pneumoniae KP-1*	10
9.	*K. pneumoniae KP-2*	11
10.	*K. pneumoniae KP-3*	12
11.	*K. pneumoniae KP-4*	11
12.	*K. pneumoniae KP-5*	11
13.	*K. pneumoniae KP-6*	8
14.	*K. pneumoniae KP-7*	11
15.	*MRSA-1*	15
16.	*MRSA-2*	16

**Table 10:** Scientific name/ strain for the organisms used in the study.

S. No.	Bacterial Strain	Gram Stain	ATCC Number
1	*Klebsiella pneumoniae-*KP1	Gram negative	ATCC 13883
2	*Escherichia coli*	Gram negative	ATCC 25922
3	*Methicillin-resistant Staphylococcus aureus* MRSA-SA1	Gram positive	ATCC BAA-41

## Data Availability

All the data has been presented in this article.
